# Measuring Effectiveness of Metamorphic Relations for Image Processing Using Mutation Testing

**DOI:** 10.3390/jimaging10040087

**Published:** 2024-04-06

**Authors:** Fakeeha Jafari, Aamer Nadeem

**Affiliations:** Department of Computer Science, Capital University of Science and Technology, Islamabad 44000, Pakistan; anadeem@cust.edu.pk

**Keywords:** image processing, metamorphic relations, metamorphic testing, mutation testing

## Abstract

Testing an intricate plexus of advanced software system architecture is quite challenging due to the absence of test oracle. Metamorphic testing is a popular technique to alleviate the test oracle problem. The effectiveness of metamorphic testing is dependent on metamorphic relations (MRs). MRs represent the essential properties of the system under test and are evaluated by their fault detection rates. The existing techniques for the evaluation of MRs are not comprehensive, as very few mutation operators are used to generate very few mutants. In this research, we have proposed six new MRs for dilation and erosion operations. The fault detection rate of six newly proposed MRs is determined using mutation testing. We have used eight applicable mutation operators and determined their effectiveness. By using these applicable operators, we have ensured that all the possible numbers of mutants are generated, which shows that all the faults in the system under test are fully identified. Results of the evaluation of four MRs for edge detection show an improvement in all the respective MRs, especially in MR_1_ and MR_4_, with a fault detection rate of 76.54% and 69.13%, respectively, which is 32% and 24% higher than the existing technique. The fault detection rate of MR_2_ and MR_3_ is also improved by 1%. Similarly, results of dilation and erosion show that out of 8 MRs, the fault detection rates of four MRs are higher than the existing technique. In the proposed technique, MR_1_ is improved by 39%, MR_4_ is improved by 0.5%, MR_6_ is improved by 17%, and MR_8_ is improved by 29%. We have also compared the results of our proposed MRs with the existing MRs of dilation and erosion operations. Results show that the proposed MRs complement the existing MRs effectively as the new MRs can find those faults that are not identified by the existing MRs.

## 1. Introduction

In the domain of computer graphics, the importance of Image Processing Applications (IPAs) is growing fast in our daily lives [1]. IPAs utilize algorithms to analyze the characteristics of an image using various methods and techniques. Digital images can be rotated, scaled, translated, and sheared by using geometric transformation. Also, in binary and grayscale images, different morphological operations such as erosion, dilation, skeletonization, and opening and closing operations are used for filtering, thinning, and pruning of the images [2].

Nowadays, IPAs are widely used in safety and mission-critical systems such as medical radiology, biometric systems, surveillance systems, etc. [1]. In medical radiology, machine learning and deep learning approaches are frequently used for automated diagnostics for patients using medical images such as MRI, CT Scan, ultrasound, etc. This diagnostic process involves some pre-processing steps, such as edge detection, and post-processing steps, such as dilation and erosion operations. Any defects in these operations will materially affect the diagnostics results. Testing of the software used in these critical systems is vital to ascertain the credibility of the results produced by these systems. Software testing is a common method to test and verify the quality of IPA software [3]. In software testing, an oracle is a mechanism that ascertains whether the software has been successfully executed for a test case or not. The Software is run for a specific test case, and the result (actual output) is compared with the anticipated result (expected output). If the output differs from what was anticipated, the program is said to be faulty [4]. Testing of IPAs is especially challenging due to the test oracle problem. For example, in image processing, edge detection is an operation that is used to compute the edges of the image. If we want to check whether the edges computed by the edge detection operator are correct or not, then we do not have the reference image (expected output) for comparison. This is the well-known oracle problem where the expected results are not obvious.

**Figure 1:**
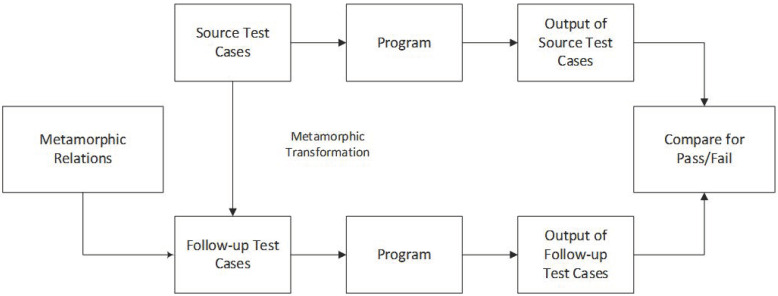
Process of metamorphic testing.

Among many solutions to test the oracle problem, metamorphic testing (MT) is the most popular technique that tackles the oracle problem in software testing of IPAs [5]. MT was first proposed by Chen et al. in 1998 [6]. In MT, we need source test cases that manifest the unexpected behavior in the system under test (SUT) [7]. The source test cases are generated through traditional test case generation techniques such as random test case generation, coverage criterion, etc. From these source test cases, a set of new test cases known as follow-up test cases are constructed using metamorphic relations (MR) [8]. MT defines some MRs, which consist of an input relation and an output relation. If the output results of the source and follow-up test cases obtained from SUT satisfy the output relation, then the program is highly reliable. Otherwise, the program will have logical errors [9]. The steps involved in MT are shown in Figure 1.

The reliability of our test results is a function of the efficacy of MT, which is dependent on the effectiveness of MRs. One of the important metrics used to evaluate MRs is the fault detection rate of that particular MR. The fault detection rate shows that either the selected test cases are able to detect faults or not (can we find violations of MRs for the corresponding test cases?) [10]. The fault detection rate is measured as the number of faults detected by the selected source test cases divided by the number of faults detected by the total number of test cases [11].

In our proposed framework, we have studied the fault detection capabilities of MRs. For the evaluation of MRs, we have initially selected four existing MRs of edge detection operation proposed by Sim et al. [12]. We have proposed six MRs for dilation and erosion operationsWe have also ascertained the fault detection capabilities of our proposed MRs (four general and two specific) for dilation and erosion operations.

The existing literature shows that Mayer and Guderlei [13] first proposed four general MRs for Euclidean distance transform. These four MRs (rotation at 90 degrees, transposition, reflection at ordinate, and reflection at abscissa) are generally applicable to all image processing operations. Furthermore, Jameel et al. [14] furthered the research by using two of these four to ascertain the fault detection rate of dilation and erosion MRs. In total, these authors have presented eight MRs (two general and six specific) for dilation and erosion operation. Jameel et al. [14] used only two general MRs, i.e., reflection at ordinate and reflection at abscissa. However, the fault detection rate of the remaining two MRs (rotation and transposition) is not determined. Therefore, we have proposed rotation and transposition MRs for dilation and erosion operations to ascertain the fault detection rate of these two MRs. The associative property is specific to the dilation operation. We have changed the order of associative property to check whether the new arrangement satisfies the dilation operation or not. This result leads us to present a new MR for dilation operation. Image translation is an operation of image processing. We have checked this operation on both dilation and erosion and come to know that it only satisfies the erosion operation. In this way, we have proposed a new MR for erosion operation.

After the selection and identification of MRs, we generated the source test cases through a criterion proposed by Jafari et al. [15]. In the paper, we (the authors) have discussed in detail how source test cases are generated using the black box testing technique (equivalence class testing) and the white box testing technique (coverage criterion). We have used 95 test cases of MRI brain images for our experiments taken from www.kaggle.com. Later, follow-up test cases are generated using source test cases and MRs. Both the source and follow-up test cases are given to the SUT. In this paper, we have the following three SUTs, i.e., edge detection, dilation, and erosion. The relation between the outputs of both the source and follow-up test cases is checked. If the MR holds between the outputs of two test cases, then the SUT has no faults; otherwise, the SUT is faulty.

Afterward, mutation testing is performed to evaluate MRs. Mutation operators always play an important role in generating the mutants. In existing literature [12,14,16,17], only a few mutation operators are used that have generated a very small number of mutants. The authors did not discuss the effectiveness of mutation operators or which operator is effective enough to generate and kill a maximum number of mutants. We have used nine mutation operators and evaluated which operator is most effective in generating and killing a maximum number of mutants.

In the mutation process, we ran the original program on source test cases and then ran the original program on the follow-up test cases. The outputs of both the test cases are recorded for comparison. In the second phase of testing, we ran the same two test cases on the mutated program. The outputs of these test cases are also recorded for comparison. If outputs of both original and mutated test cases satisfy their related MR, then it shows that the mutant is not killed; otherwise, the mutant is killed. Afterward, the mutation score is calculated to check the fault detection rate of each MR. If the mutation score is near 1, then it shows that the MR is strong, or else the MR is weak enough to find the violation.

This paper makes the following contributions:

- We have proposed six new MRs for dilation and erosion operations and ascertain the effectiveness of these MRs while also assessing improvements in them using mutation testing.
- We have compared our six proposed MRs with the eight existing MRs for dilation and erosion operations.
- In existing literature, only two mutation operators are used for the evaluation of edge detection and morphological image operations. We have used nine mutation operators to improve the effectiveness of edge detection and morphological image operation (dilation and erosion) MRs. We have also compared the result of our proposed framework with the existing techniques.
- We have also checked the effectiveness of mutation operators to determine which operator is more effective in generating and killing a maximum number of mutants.

This paper is organized as follows; Section 2 discusses the related work. Section 3 describes the existing and newly proposed MRs. Section 4 discusses the proposed framework for the evaluation of MRs. In Section 5 experiment design is narrated. Section 6 discusses the results and discussion whereas Section 7 describes the conclusion.

## 2. Related Work

In literature review, we have covered those papers where MRs are evaluated to improve the effectiveness of MT. MT is a common technique to improve the test oracle problem where it is hard to assess the output correctly when an arbitrary input has been given to the SUT [18].

Many researchers have used different image processing operators, such as edge detection, image region growth, dilation and erosion, and used their properties as metamorphic relations. The effectiveness of these MRs is checked through mutation testing. Sim et al. [12] proposed a framework to determine the effectiveness of MT. To conduct the experiments, collections of images are needed for the generation of test inputs. Unlike model-generated images, camera-captured images (real images) from published image libraries are selected randomly. Mutation testing is used to evaluate the fault detection rate of MT. Single operator faults and stride implementation faults are seeded into the Sobel edge detection program. In single-operator faults, two types of operators are used: logical operator replacement (LOR) and relational operator replacement (ROR). Results show that MT is capable of detecting faulty edge detection programs up to 90%. Jameel et al. [14] discussed the oracle problem in IPAs and showed how SUT properties could be used as MR. The authors have studied some properties of morphological image operations. The effectiveness of MRs can be analyzed through mutation testing. In order to conduct the experiments, input images are selected randomly. Mutation testing is used to show the effectiveness of the above-mentioned MRs. Therefore, errors are deliberately added to the Mex C code. The mutation score tells the number of killed mutants. The mutant is said to be killed if an MR is able to detect the bug. It is concluded that for bug identification, specific images are needed instead of general input images such as Lena. Jiang et al. [19] applied MT to the image region growth program. Mutation testing is used to find the effectiveness of MRs. In this paper, MT is applied to test the aerospace image processing software. A segmental symbolic evaluation method is used to generate the input data. The original program implemented in C language is executed sequentially with three mutant programs. The program is said to be faulty if an MR violation can be seen after the validation of output relations.

Many researchers have been fascinated by the use of MT techniques in machine learning algorithms as well. Jameel et al. [20] used support vector machine (SVM) to automate the interpretation of the output results of test oracle requirements. These authors have designed a comparative study to gauge the effectiveness of their proposed scheme against the latest MT oracle technique and the traditional statistical oracle method. Thirty-five distinctive errors are introduced to the original program written in C language to create 35 unique resultant programs. For evaluation purposes, these authors have created the output images from these 35 versions of the image dilation program for pass or fail criteria. Half of the selected images are used to train SVM using various features (wavelet features, binary features, hough features, statistical features) of dilated images to analyze their effectiveness. The results confirmed that SVM was better in terms of the lowest classification error than the other two techniques. Chan et al. [21] integrated the pattern classification technique with MT. A trained classifier (C4.5) is employed for the test oracle by labeling pass/fail. The passed test outputs may also show false positive/negative failures, which are then processed for additional testing. This proposal has proven to be efficient and effective.

MT techniques have also been used with structural testing. Ding et al. [17] used a discrete dipole approximation program (ADDA) implemented in FORTRAN and C++ to check the effectiveness of MT. In this paper, statement coverage is used to check the effectiveness of test cases, whereas mutation testing is used to check the effectiveness of MT. Due to the unknown test output relations, the MRs of this program are considered weak and inadequate. Ding and Hu [16] developed a method for the adequacy of MRs. Coverage criterion, mutation analysis, and mutation tests for testing MRs are critical factors in evaluating the adequacy of MRs. An image processing program that is used to reconstruct a 3D biological cell is used to explain the author’s proposed theory. A case study is performed using a complex Monte Carlo program to gauge the effectiveness of this proposed framework. The results prove the utility of their proposed method for the testing of other scientific software as well. Table 1 shows the summary of related work.

After studying the literature, some of the research gaps are identified and are given below:

- In literature, test cases are selected and generated randomly. Random selection leads to an unfair distribution of parametric values, which ultimately affects the testing process.
- In existing techniques, MR evaluation is conducted through mutation testing. This evaluation is not comprehensive, as only a few mutation operators are used to check the fault detection rate of MRs. The total number of mutants generated through these mutation operators is quite low which makes the testing weak.
- In existing literature, no work has been conducted to check the effectiveness of mutation operators. It is not highlighted that which operator is more valuable to generate and kill maximum number of mutants.

## 3. Metamorphic Relations

In MT, the central element is the set of MRs, which are the necessary properties of the SUT or the algorithm [22]. MR plays a significant role in MT as it validates the relations between the test outputs of a program having a test oracle problem. Generally, MR is the property of a function (f) having inputs x_1_, x_2_, x_3_, … x_n_, where *n* is greater than 1. Their corresponding outputs are f(x_1)_, f(x_2)_, f(x_3)_, … f(x_n_) [23]. The identification of MRs requires expert knowledge in the field of Image Processing (IP) as well as guidance provided by the experiences (Mayer et al. [13], Jameel et al. [14]).

In this paper, we have worked on the MRs of edge detection and two morphological image operations, i.e., dilation and erosion.

### 3.1. MRs for Edge Detection

We have used the MRs of edge detection proposed by Sim et al. [12]. The complete details of these MRs are given in [12,15]. The MRs are shown in Table 2.

Table 2 shows the MRs for edge detection where E is the Sobel edge detection, and Im is the input image.

### 3.2. MRs for Dilation and Erosion

In this section, we have described the existing and proposed MRs for the dilation and erosion operations.

#### 3.2.1. Existing MRs for Dilation and Erosion

The existing MRs proposed by Jameel et al. [14] for dilation and erosion operations are given in Table 3.

Table 3 shows the MRs for dilation and erosion operations. The details of these MRs are given in [14].

#### 3.2.2. Proposed MRs for Dilation and Erosion

We have proposed six new MRs for dilation and erosion operations. Our proposal consists of four general and two specific MRs of dilation and erosion. In Table 4, we have discussed our proposed MRs with their mathematical properties. The details of these MRs are given below:

## 4. Evaluation of Metamorphic Relations

In this section, we have discussed the historical evaluations of MRs from the existing literature. We have also suggested a new method to evaluate MRs in our proposed framework.

The evaluation of MRs in MT involves assessing how well these relations can effectively guide the generation of additional test cases and help verify the correctness of a software system. The source and follow-up test cases are executed, and their outputs are verified against the relevant MRs, of which any violation implies that the software under test is faulty. The MRs are considered strong and do not satisfy the relation easily. The higher the number of test cases that satisfy the MR, the weaker the MR. Suppose we have a program P that computes the sin function. There are two MRs to compute the sin function.

MR_1_: *sin*
*x* = *sin* (180 − *x*)

MR_2_: *sin*
*x* = *sin* (*x* + 360)

The MR that satisfies the relation on maximum test cases is considered weak and vice versa. For all positive values of *x*, MR_1_ is better than MR_2_ as MR_2_ satisfies the relation for all positive values, and thus, MR_2_ will always be a weak MR.

The evaluations of MRs based on a random selection of source test cases using existing techniques are not comprehensive with respect to the truly random generation of source test cases. The available sample population from which random source test cases are selected is missing many types of image characteristics such as dimension, resolution, bit depth, type of image, etc.

Sim et al. [12] and Jameel et al. [14] selected the images randomly from various published image libraries available online. Each library has a set of images with only one or two image characteristics. If we create a new library using all the images from these libraries, many image properties will still be missing. By selecting the test cases randomly from these libraries, it is probable that all the selected test cases may cover only one property of the image while ignoring the others. This will definitely affect the testing process because of the lack of diversity in test images.

**Figure 2:**
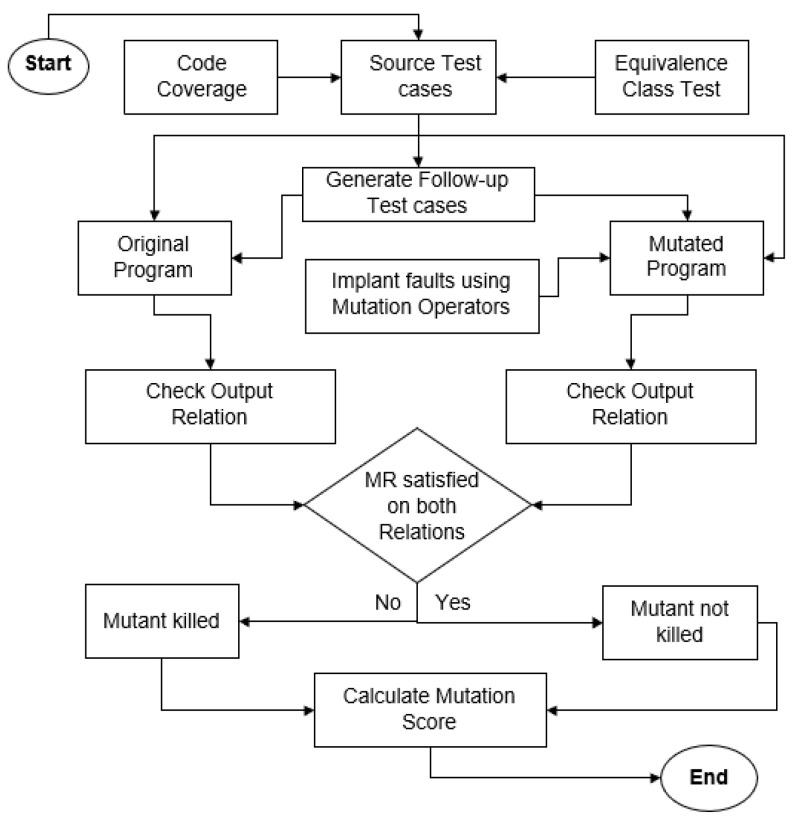
Evaluation process of MRs.

Furthermore, in existing literature, mutation testing is used for the evaluation of MRs. As discussed earlier, mutation operators play a dominant role in generating a significant number of mutants. In existing techniques, Sim et al. [12] used two mutation operators, i.e., ROR and LOR, whereas Jameel et al. [14] used only one mutation operator (ROR) for the generation of mutants. These two operators only produce a maximum of 33 mutants, which is not a significant number for the purpose of evaluation. So, more mutation operators should be needed for the extensive evaluation of MRs. Figure 2 shows the evaluation process of our proposed framework.

### 4.1. Source Test Case Generation

The foremost step is the generation of source test cases (original test cases). In literature, source test cases are generated either through some traditional test case generation techniques as discussed earlier or through some tool such as EvoSuite (it generates source test cases automatically through coverage criterion) [5]. Nowadays, few researches are emerged in the direction of generation and selection of source test cases that are effective in fault detection [24].

As discussed in the literature, the general source test case generation criterion is to generate the test cases randomly. It is a probability that randomly generated test cases may cover only one characteristic of the image while ignoring the other ones. Sim et al. [12] also suggested that considering the characteristics of the image can improve the mutation score of MRs. Keeping this in mind, we have proposed a source test case generation criterion in our previous paper. In this criterion, we have generated the source test cases through equivalence class testing and coverage criterion. In equivalence class testing, we have considered the attributes of images and grouped them into five distinct classes such as horizontal dimension, vertical dimension, resolution, bit depth, and image type. The details of the formation of source test cases are given in [15]. We have used the same (95) test cases for our experiments. Afterward, the adequacy of source test cases is checked through program coverage (statement coverage and branch coverage). If the test cases (accumulatively) do not achieve 100% coverage, then we need more test cases to achieve 100% branch coverage.

### 4.2. Metamorphic Testing

In IP, it is hard to test a program without a test oracle, as the outcome is not predictable. In MT, we can detect that either the test case has passed or failed by generating new test cases for further evaluation. The steps of MT are discussed in Figure 1. In MT, MR transforms the existing source test cases into new test cases known as follow-up test cases [25]. When the follow-up test cases are generated, both test cases are given to the SUT. By executing the SUT, the output data is generated for each test case [23]. Processing of SUT is conducted by comparing the output relation of source and follow-up test cases. The satisfaction of MR shows that the SUT is not faulty, whereas the dissatisfaction of MR shows that the SUT is faulty.

### 4.3. MR Evaluation Using Mutation Testing

Evaluation of MRs is an indication of their fault detection capabilities. The greater the fault detection capabilities, the greater the ability to detect more faults from a program. We have checked the fault detection rate of these MRs through mutation testing.

Mutation operators play an important role in generating mutants in mutation testing. Previously, very few mutation operators were used, which did not even highlight the effectiveness of these operators. We have used nine mutation operators to check which operator is most effective for generating and killing a significant number of mutants. Our work is relevant to [12,14], so we have compared the mutation operators used in our proposed framework with these two techniques. The mutation operators used in the existing techniques [12,14] and in the proposed framework are given in Table 5.

If the output of source and follow-up test cases satisfies the relation, then mutation testing can be performed by seeding the faults into the original program to check for MR violation. The process of checking the original and mutated program is explained through an example. Suppose we have two test cases, t_1_ (source test case) and t′_1_ (follow-up test case), that have to be tested under the original program p. The outputs of tests t_1_ and t′_1_ can be recorded as r_1_ and r′_1_. Afterward, the same test cases, t_1_ and t′_1_, can be run on the mutated program p′ with mutant m. Record the outputs as r_2_ and r′_2_. If both (r_1_, r′_1_) and (r_2_, r′_2_) satisfy their related MR, then the mutant m is not killed [19]. Otherwise, the mutant is killed. After mutation testing, the mutation score is to be calculated. The mutation score indicates the fault detection rate of each MR. In the existing literature, i.e., [12,14,16,19], all the MRs are evaluated by seeding faults manually in the code or through a tool that calculates the mutation score automatically through the traditional mutation testing approach. We have seeded the faults manually and checked the relation manually on both the programs (original and mutated) for the calculation of the mutation score.

The mutation score indicates the fault detection rate (FDR) of MR. For the calculation of the mutation score, we have examined the mutants manually and have removed all the equivalent mutants. The formula for the mutation score is given below:

(1) $$FDR=\frac{Number\;of\;killed\;Mutants}{Total\;Number\;of\;Non\text{-}equivalent\;Mutants}\times 100\%$$

According to the formula, if the mutation score is 1 then it means that MR is strong (high fault detection rate) whereas a 0 score would show MR is weak (low fault detection rate). We can also say that if the mutation score is near 0, the MR is weak enough to find the violation, while on the other hand, if the mutation score is near 1, the MR is strong.

### 4.4. Advantages of Proposed Framework

- In the proposed fraework, we have used nine applicable mutation operators to ascertain the effectiveness of MRs. The higher the number of mutation operators used, the higher the number of faults detected. The use of these operators contribute to the improvement of software quality, the effectiveness of test suites, and improvement in test coverage.
- We have proposed new MRs in the field of IP. Overall, proposing new metamorphic relations in image processing contributes to the advancement of testing methodologies, algorithm validation techniques, and research practices, ultimately leading to more reliable, robust, and efficient image processing systems and applications.

## 5. Experiment Design

In this section, we have discussed the details of SUT used for our experiment: source code, dataset, original test cases, coverage criterion, and mutation operators used.

### 5.1. Proposed Evaluation

The subject programs in this paper consist of the following:

- Sobel edge detection program
- Dilation and erosion programs

**Figure 3:**
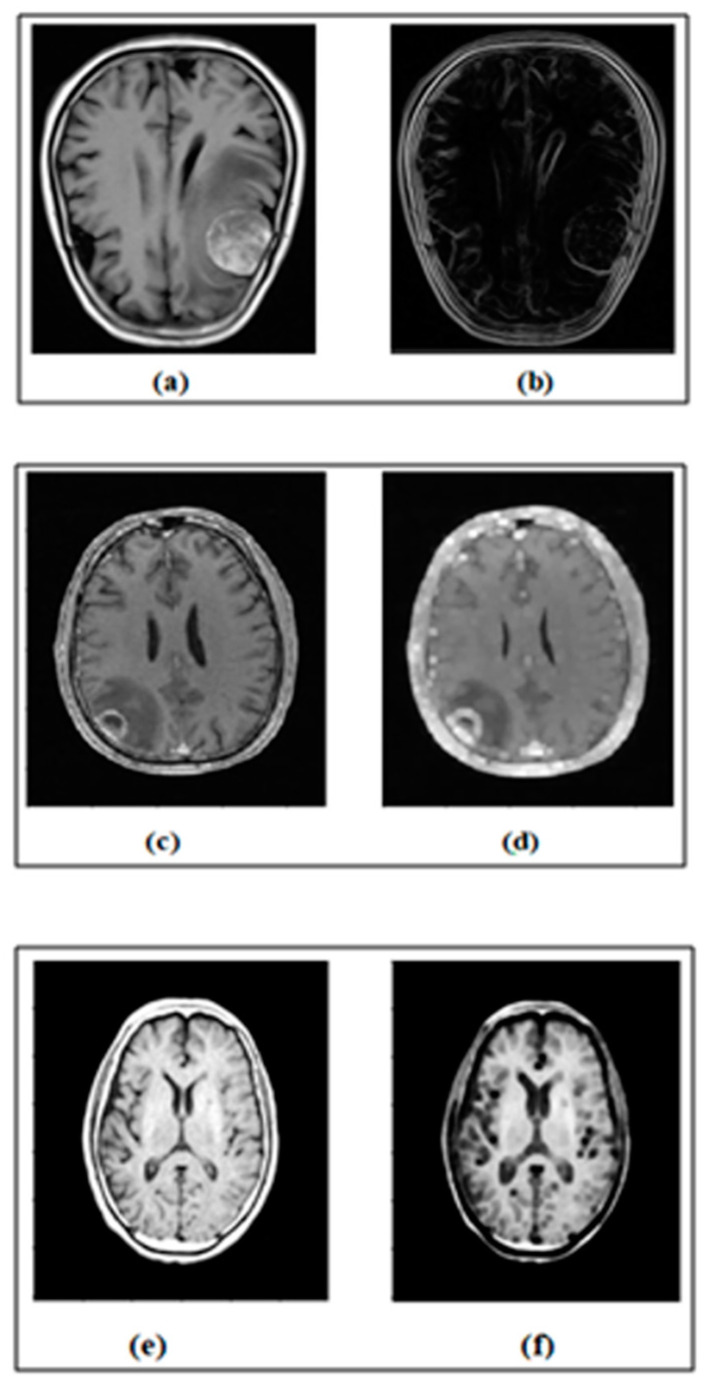
(**a**) Input image. (**b**) Output of edge detection. (**c**) Input image. (**d**) Output of dilation. (**e**) Input image. (**f**) Output of erosion.

The properties of edge detection and morphological operations are used as MRs. In IP, edge detection plays a vital role in identifying the immediate changes in grayscale images. Identifying the edges of the images can be invaluable for different real-world applications [26]. Similarly, dilation and erosion are the main morphological operations that increase or decrease the region of the image according to the structuring element [2]. The inputs and outputs of the edge detection program, a dilation and erosion program, are given in Figure 3.

Figure 3 shows the sample inputs of MRI brain images and their expected output images after performing edge detection, dilation operation and erosion operation.

### 5.2. Source Code

We have used a well-structured code of Sobel edge detection and dilation and erosion operations written in Python version 3.8.3 for our implementation. The code of edge detection consists of 41 statements and 10 branches. Similarly, the code of dilation and erosion consists of 46 statements and 12 branches, respectively. The sources of the above codes are given in Table 6.

#### 5.2.1. Pseudocode of Dilation/Erosion Operation

FUNCTION dilation(image, kernel_size, kernel):
n, m = shape_of(image)
transpose_kernel = transpose(kernel)
create empty array edges_img with shape (n, m)
x = y = 0
WHILE y < m + 1 − kernel_size DO
IF x < n + 1 − kernel_size THEN
local_pixels = get_subarray(image, x, y, kernel_size)
val = minimum(local_pixels + kernel)
IF val > 255 THEN
set edges_img[x + (kernel_size//2), y + (kernel_size//2)] = 255
ELSE IF val < 0 THEN
set edges_img[x + (kernel_size//2), y + (kernel_size//2)] = 0
ELSE
set edges_img[x + (kernel_size//2), y + (kernel_size//2)] = maximum/minimum(local_pixels)
END IF
increment x by 1
ELSE
increment y by 1
set x to 0
END IF
END WHILE
Convert edges_img to uint8 data type
RETURN edges_img
END FUNCTION
FUNCTION display(images):
titles = [‘original’, ‘After Dilation’]
FOR i FROM 1 TO length_of(images) DO
CREATE a new figure
SET title of the figure to titles[i]
DISPLAY the i-th image with gray colormap
END FOR
SHOW all figures
END FUNCTION
SET path to image file path
LOAD image from path
images = [image]
APPEND dilation(image, 5, ones(5,5)) to images
DISPLAY images

#### 5.2.2. Pseudocode of Edge Detection Operation

FUNCTION sad():
vertical_filter = [[−1,−2,−1], [0,0,0], [1,2,1]]
horizontal_filter = [[−1,0,1], [−2,0,2], [−1,0,1]]
path = “path_to_image.jpg”
img = read_image(path)
n, m, = dimensions_of_image(img)
edges_img = copy_of(img)
x = y = 0
WHILE y < m + 1 − 3 DO:
IF x < n + 1 − 3 THEN:
local_pixels = get_local_pixels(img, x, y)
vertical_transformed_pixels = vertical_filter * local_pixels
vertical_score = sum_of_elements(vertical_transformed_pixels)/4
horizontal_transformed_pixels = horizontal_filter * local_pixels
horizontal_score = sum_of_elements(horizontal_transformed_pixels)/4
edge_score = square_root_of((vertical_score^2) + (horizontal_score^2))
edges_img[x + 1, y + 1] = [edge_score] * 3
x = x + 1
ELSE:
y = y + 1
x = 0
END IF
END WHILE
edges_img = edges_img/edges_img.max()
display_image(edges_img)
save_image(‘output_path.jpg’, edges_img)
IF _name_ == “_main_”:
sad()

### 5.3. Dataset

**Figure 4:**
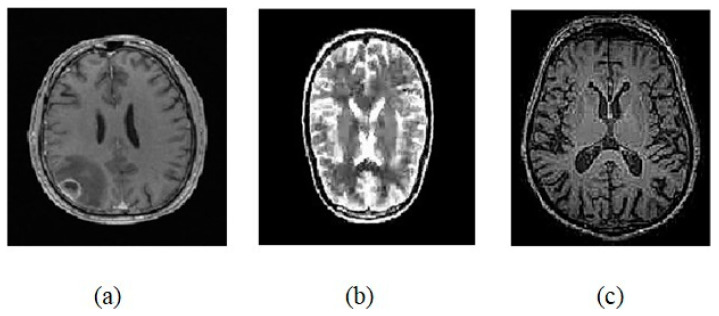
(**a**) T1 Weighted image. (**b**) T2 Weighted image. (**c**) Flair image.

For our study, a diversified collection of MRI brain images is taken from https://www.kaggle.com/datasets/abhranta/brain-tumor-detection-mri?resource=download (access on 17 February 2024). The dataset consists of 1500 images having brain tumors and 1500 images having no brain tumors. The basic three types of images used as test cases are shown in Figure 4.

### 5.4. Source Test Cases

We have selected 95 source test cases through black box testing technique (strong equivalence class testing) and coverage criteria (statement coverage and branch coverage); a criterion proposed in our previous paper [15].

### 5.5. Source Test Cases

The coverage of source test cases is checked through statement coverage and branch coverage, respectively. The coverage detail is given in Table 7.

As shown in Table 7, 95 test cases (accumulatively) cover 100% code coverage in all three programs, so we do not need more test cases for our test suite.

### 5.6. Mutation Operators

Mutation operators cover a wide range of potential faults or mutations that can occur in the code. They encompass various types of changes that may affect the behavior of the program. Each mutation operator targets a specific kind of fault. For example, some operators might mutate arithmetic operators, while others might mutate relational operators or logical operators. For our study, the mutation operators used in Python language are taken from GitHub—mutpy/mutpy: MutPy is a mutation testing tool for Python 3.x source code. There are twenty traditional and seven experimental operators. The list of traditional and experimental operators is as follows:

AOD—arithmetic operator deletion
AOR—arithmetic operator replacement
ASR—assignment operator replacement
BCR—break continue replacement
COD—conditional operator deletion
COI—conditional operator insertion
CRP—constant replacement
DDL—decorator deletion
EHD—exception handler deletion
EXS—exception swallowing
IHD—hiding variable deletion
IOD—overriding method deletion
IOP—overridden method calling position change
LCR—logical connector replacement
LOD—logical operator deletion
LOR—logical operator replacement
ROR—relational operator replacement
SCD—super calling deletion
SCI—super calling insert
SIR—slice index remove

**Experimental mutation operators:**

CDI—class method decorator insertion
OIL—one iteration loop
RIL—reverse iteration loop
SDI—static method decorator insertion
SDL—statement deletion
SVD—self variable deletion
ZIL—zero iteration loop

The use of mutation operators is dependent on the source code of a program. We have used all the mutation operators that are applicable according to our source code. The mutation operators used in this research are given below:

AOD → Unary arithmetic operator deletion
AOR → Arithmetic operation replacement
LOR → Logical operator replacement
ROR → Relational operator Replacement
OIL → One iteration loop
RIL → Reverse iteration loop
SIR → Slice index remove
SDL → Statement deletion
ZIL → Zero iteration loop

## 6. Results and Discussions

In this section, we will discuss the MR evaluation results of our testing methodology in detail.

### 6.1. Effectiveness of Mutation Operators

In this section we have assessed the effectiveness of mutation operators by calculating the percentage of mutants generated and mutants killed. Mutation score of each operator shows the FDR of each mutation operator. The formula of mutation score depicted in Equation (1) is used to calculate the fault detection rate of each mutation operator. The percentage of generated mutants is calculated by the formula given in Equation (2).

(2) $$M\;generated=\frac{No.\;of\;M\;generated\;by\;each\;op*100}{Total\;no.\;of\;M\;generated\;by\;all\;the\;op}$$

In Equation (2), mutant is denoted by “M” and mutation operator is denoted by “op”.

#### 6.1.1. Effectiveness of Mutation Operators Used in Edge Detection

Table 8 shows the effectiveness of mutation operators in terms of mutants generated and mutants killed by each operator for edge detection.

In the existing technique of Sim et al. [12], a total of 31 mutants have been generated by using only two mutation operators, i.e., ROR and LOR. In our proposed framework, we have employed nine mutation operators to generate a total of 162 mutants. It is observed that mutation operators such as AOD, COI, ZIL, and SIR have shown 100% mutation scores in all four MRs. But their percentage with respect to generated mutants is very low, i.e., 3.70%, 1.23%, 2.46%, and 1.23%, respectively. The effectiveness is dependent on two factors, i.e., mutation score and number of mutants generated. The operator that scores a high percentage in one of the two factors and scores very low in the other is not as effective as the one having moderate scores in both factors. So, the AOR operator is the most effective operator because its lowest mutation score is 63% (MR_4_), and the highest mutation score is 74% (MR_1_), whereas its percentage to generate mutants is 60.49%. RIL is the least effective operator because its lowest mutation score is 0% (MR_4_), and the highest mutation score is 100% (MR_2_), whereas its percentage to generate mutants is only 1.23%. SDL operator has achieved a good mutation score of 81.25%, followed by ROR having a mutation score of 73.33% against each MR, whereas their percentage to generate mutants is 9.87% and 18.51%, respectively. The effectiveness of SDL and ROR is almost similar because the mutation score of SDL is 12% higher than ROR, whereas the percentage of the ROR operator in terms of mutation generation is 10% higher than the SDL operator.

#### 6.1.2. Effectiveness of Mutation Operators Used in Dilation and Erosion

Now, we will discuss the effectiveness of mutation operators used in dilation and erosion operations for the proposed MRs. Table 9 shows the FDR (mutation score) and percentage of mutants generated by each mutation operator used in dilation and erosion operations.

In the literature, Jameel et al. [14] used only one mutation operator for the generation of mutants and created only 33 mutants, whereas we used eight mutation operators and produced 130 mutants in total. It is observed from Table 9 that the AOR operator is the most effective operator because it has generated a maximum number of mutants, i.e., 95, and its fault detection rate is greater than 50% against each MR. While the FDR of the ZIL operator is 100%, it has generated only one mutant and has a percentage of 0.76. ROR has a better percentage of generating the mutants, i.e., 15% and also, a mutation score lies between 15 to 30%. It is concluded from this section that the AOR operator is the most effective operator in terms of mutants killed and mutants generated in both the subject programs of edge detection and dilation and erosion.

### 6.2. Effectiveness of Metamorphic Relations

The effectiveness of MRs is determined through mutation testing. FDR depicted in Equation (1) shows the effectiveness of each MR. We have assessed the effectiveness of existing MRs (edge detection and dilation and erosion) and proposed MRs (dilation and erosion).

#### 6.2.1. Effectiveness of Edge Detection MRs

The fault detection rate (mutation score) defines the strength of each MR. FDR is calculated through mutation testing. The FDR of edge detection MRs is given in Table 10.

We have generated a total of 162 mutants for edge detection manually by introducing one fault at a time. It is observed from Table 10 that MR_2_ has killed a maximum number of mutants, i.e., 126, followed by MR_1_, which has killed 124 mutants. MR_3_ and MR_4_ have killed the same number of mutants, i.e., 112 mutants each. The last column in Table 10 shows the FDR (in percentage) of each MR.

#### 6.2.2. Effectiveness of Dilation and Erosion MRs

The FDR of existing operations of dilation and erosion using our proposed framework are given in Table 11.

We have generated a total of 130 mutants for dilation and erosion operation. Table 11 shows that R_1_ has killed a maximum number of mutants, i.e., 70, followed by R_2_ and R_7_, with 68 killed mutants each. R_6_ has killed the least number of mutants, i.e., 45. After calculating the FDR, it is observed that R_1_ has the highest FDR of 53.84%, thus making R_1_ the most effective MR. R_2_ and R_7_ have the second-highest FDR of 52.30% each. R_6_ has the lowest FDR of 34.61%, thus making this MR least effective.

#### 6.2.3. Effectiveness of Proposed MRs

As discussed earlier, for the dilation and erosion operation, we have suggested six MRs. Four of the six MRs are general and can be used for the majority of IP operations, while the remaining two MRs are particular to dilation and erosion operations. We have also assessed the effectiveness of our proposed MRs using our proposed framework. The FDR of proposed MRs are given in Table 12.

Table 12 shows that we have generated a total of 130 mutants. It is observed that MR_6_ has the highest FDR, killing 68 mutants, followed by MR_4_, which killed 65 mutants. MR_1_ and MR_3_ have killed 59 mutants each. The FDR of MR_2_ is the lowest because it has killed 57 mutants. It is observed that the FDR of our proposed MRs are neither too high nor too low but are significant enough to find the violations in all the respective MRs. However, the most effective MR among the proposed MRs is MR_6_ (image translation), which has the highest FDR of 52.30%, followed by MR_4_ (transposition in erosion operation) with an FDR of 50%. MR_2_ (counterclockwise rotation at 90 degrees in erosion operation) is the least effective, with an FDR of 43.84%.

### 6.3. Comparison of Proposed Framework with Existing Techniques

We have compared the results of our proposed framework with Sim et.al [12] and Jameel et al. [14]. Table 13 shows the statistics of existing techniques and proposed framework.

According to the statistics given in Table 13, Sim et al. [12] selected 30 images as source test cases from different image libraries given in [12]. The images used by the authors are very limited and not diverse in nature. The Kodak site has 24 images, and the image compression site has only 15 images. All the images have the same bit depth of 24 and resolution of 96 dpi. All the images have only two dimensions, 768 by 512 or 512 by 768 (Kodak site) and a single dimension of 700 by 525. Jameel et al. [14] have not mentioned the source as well as the number of test cases selected for their experiments. We have used the data set of MRI brain images taken from Kaggle.com. The dataset is comprised of 3000 images with diverse image properties. From 3000 images, we have selected 95 images using equivalence class testing and code coverage. Sim et al. [12] used two mutation operators and generated only 31 mutants. Jameel et al. [14] used only one operator and generated 33 mutants. We have used nine mutation operators in the edge detection program and eight operators in the dilation and erosion program to generate 162 and 130 mutants, respectively.

#### 6.3.1. Comparison Results of Edge Detection

We have compared the results of our proposed framework with Sim et al. [12]. The comparison results are given in Table 14.

Table 14 shows that in the existing technique of Sim et al. [12], MR_2_ has the highest FDR followed by MR_3_, whereas in the proposed framework, MR_2_ has the highest FDR followed by MR_1_. In the existing technique, the FDR of MR_1_ and MR_4_ are the same, i.e., 45%, whereas in the proposed framework, the FDR of MR_3_ and MR_4_ are the same, i.e., 69.13%. In the proposed framework, the FDR of MR_1_ and MR_4_ is far better than the existing technique, i.e., 76.54% and 69.13%, respectively. The FDR of MR_2_ and MR_3_ is also improved by 1%.

#### 6.3.2. Comparison Results of Dilation and Erosion

Jameel et al. [14] evaluated eight MRs of dilation and erosion operation. We have also evaluated the same MRs using our proposed framework. The comparison results are given in Table 15.

Table 15 shows that out of eight MRs, four MRs, i.e., R_1_, R_4_, R_6_, and R_8_, have improved FDR using our proposed framework. The FDR of R_1_ (53.84%), R_6_ (34.61%), and R_8_ (50%) are far better than the existing technique, having a FDR of 15%, 18%, and 21%, respectively. In existing techniques, R_2_, R_3_, R_5_, and R7 have high FDR, i.e., 58%, 97%, 50% and 73%, because they have used only one mutation operator and considered only one type of fault. In the proposed framework, the FDR of all the MRs is moderate, neither too high nor too low, thus making it effective to find the violations in all respective MRs. In the proposed framework, R_1_ is improved by 39%, R_4_ is improved by 0.5%, R_6_ is improved by 17%, and R_8_ is improved by 29%. The FDR of some of the MRs in the existing technique is high because the number of mutants generated is just 31.

### 6.4. Comparison Results of Proposed MRs with Existing MRs of Dilation and Erosion

In this section, we compare the results of our proposed MRs with those of the existing MRs. As described earlier, we have used eight mutation operators for the evaluation of dilation and erosion MRs and have generated 130 mutants in total. The number of mutants against each mutation operator is depicted in Table 16.

In mutation testing, we have used eight mutation operators for the evaluation of dilation and erosion MRs. In the AOR operator, we have used six types of faults such as addition (+), subtraction (−), multiplication (*), division (/), exponent/power (**), and floor division (//). It is observed that there are nine arithmetic faults (collectively) that are identified by the proposed MRs and are not identified by any of the existing MRs. Among these faults, eight faults are identified by MR_4_, five faults are identified by MR_5_, and nine faults are identified by MR_6_. So, we can say that MR_4_, MR_5_, and MR_6_ are more effective operators than MR_1_, MR_2_, and MR_3_ because they have identified additional faults not identified by any of the existing MRs. We have observed the presence of alive mutants in both existing and proposed MRs. So, by combining both the MRs, the total number of alive mutants was reduced. Hence, it is concluded that the proposed MRs complement the existing MRs effectively.

## 7. Conclusions

Testing of Image Processing Applications (IPAs), of course, is a challenging task because of the absence of test oracle. Metamorphic testing is an efficient method to deal with the applications with a test oracle problem. Metamorphic relations play an important role in metamorphic testing. A metamorphic relation relates two or more inputs with their expected outputs after execution of the properties of the target program. Properties of different image processing operations can also be used as metamorphic relations.

In this research, we have proposed six new MRs for morphological image operations (dilation and erosion). The fault detection rate of newly proposed MRs, along with existing MRs, is determined through mutation testing. The effectiveness of mutation operators is also determined by which operator is more effective in generating a maximum number of mutants and through which operator a maximum number of mutants are killed. AOR is considered the best operator in both the subject programs as it generates and kills a maximum number of mutants. We have compared the results of our proposed approach with the existing techniques of edge detection and morphological image operations. Our results demonstrate that the mutation score of all the MRs of edge detection has improved, whereas the MRs of dilation and erosion have shown improvement in four MRs (out of 8). While comparing our proposed MRs with the existing MRs of dilation and erosion operations, we have come to the conclusion that the proposed MRs complement the existing MRs effectively as the proposed MRs are able to find those faults that are not identified by the existing MRs.

Some of the limitations of this study are:

- In this research, we have used only one dataset for the experiments. We can use more datasets for convincing testing.
- One of the limitations of this research is the use of only one algorithm for edge detection. There are several other edge detection algorithms that can be used to make the experiments more substantial.
- We have used only one programming language, i.e., Python. We could use more languages, such as Java, C++, etc., so that more mutation operators can be used.

The future directions of this research are:

- In this research, statement coverage and branch coverage are used for code coverage. In the future, the proposed research for the evaluation of MRs can be strengthened by using more coverage criteria for improved coverage. The future work involving coverage criterion may include multiple condition coverage (MCC), where every combination of conditions’ outcomes is tested at least once in a decision; modified condition/decision coverage (MCDC) where a decision’s potential outcomes are determined by each condition contained within the decision, all def-use (definition usage) coverage where all-def coverage is attained when all defs of any variable are covered and all-uses coverage is attained when a path from each def to each use of that def has been exercised, etc.
- The research can also be strengthened by using additional mutation operators that will cover the fault types not used in the proposed research, such as logical connector replacement (LCR), break continues replacement (BCR), constant replacement (CRP), class method decorator insertion (CDI), etc.

## Figures and Tables

**Table 1:** Summary of related work.

Ref Papers	SUT	Image Generation Method	Testing Method	Mutation Operators
Sim et al. [12]	Sobel Edge Detection Program	Randomly selected from published image libraries	Mutation Testing	LOR, ROR
Jameel et al. [14]	Dilation and Erosion Programs	Randomly Selected	Mutation Testing	ROR
Jiang et al. [19]	Image Region Growth program	Segmental symbolic evaluation method	Mutation Testing	Milu operators
Jameel et al. [20]	Dilation Program	Ground truth images are chosen randomly	Mutation Testing	ROR
Chan et al. [21]	Mesh Simplification Program of Image Rendering	Randomly Selected	Mutation Testing	MuJava operators
Ding et al. [17]	Discrete Dipole Approximation Program	Random Generation	Structural Testing and Mutation Testing	ABS, ROR
Ding et al. [16]	Monte Carlo Program and 3D Structure Reconstruc-tion Program	Random or Category Based Selection	Structural Testing and Mutation Testing	Modify coefficient, add constant, AOR, COR, CRP, SDL

**Table 2:** MRs for edge detection.

MR	Mathematical Property
MR_1_: Counter clock wise rotation at 90 degree	C(E(Im)) = E(C(Im))
MR_2_: Transposition	T(E(Im)) = E(T(Im))
MR_3_: Reflection at the ordinate	M_x_(E(Im)) = E(M_x_(Im))
MR_4_: Reflection at abscissa	M_y_(E(Im)) = E(M_y_(Im))

**Table 3:** Existing MRs for dilation and erosion.

MR	Mathematical Property
R_1_: Reflection at the ordinate	${Ref}_{ord}\left(Output\left(I\right)\right)=Output({Ref}_{ord}\left(I\right))$
R_2_: Reflection at abscissa	${Ref}_{ord}\left(Output\left(I\right)\right)=Output({Ref}_{ord}\left(I\right))$
R_3_: Duality	$\delta_{s}(I)={ℇ}_{s}(I$^c^) ${ℇ}_{s}(I)=\delta_{s}(I$^c^) where c is the complement of an image I.
R_4_: Non-inverses	$\delta_{s}\left({ℇ}_{s}\left(I\right)\right)\neq I\neq {ℇ}_{s}\left(\delta_{s}\left(I\right)\right)$
R_5_: Size of image object changes	${Size}_{obj}\left(\delta_{s}\left(I\right)\right)\geq {Size}_{obj}\left(I\right)\;and\;{Pix}_{list}I\subset {Pix}_{list}\delta_{s}\left(I\right)$
R_6_: No. of objects in image changes	${Number}_{obj}\left(\delta_{s}\left(I\right)\right)\leq {Number}_{obj}(I)$
R_7_: Commutative	$\delta_{s}\left(I\right)=I⊕S=S⊕I=\delta_{I}\left(S\right)$ ${ℇ}_{s}\left(I\right)\neq {ℇ}_{I}(S)$
R_8_: Translation invariance	$\delta_{s+x}\left(I\right)=\delta_{s}\left(I\right)+x$

**Table 4:** Proposed MRs for dilation and erosion.

Proposed MRs	Explanation
**Counter clock wise rotation at 90 degree** ${\mathrm{MR}}_{1}:C\left(\delta_{s}\left(Im\right)\right)=\delta_{s}(C\left(Im\right))$ ${\mathrm{MR}}_{2}:C\left({ℇ}_{s}\left(Im\right)\right)={ℇ}_{s}(C\left(Im\right))$	Where, *Im* is the input Image, *C*(.) is the counter clockwise rotation at 90 degree, $\delta_{s}$ is the dilation and ${ℇ}_{s}$ is the erosion operation. The image output of counter-clock wise rotation at 90 degree followed by morphological operations should be similar to image output of morphological operations followed by counter-clock wise rotation at 90 degree.
**Transposition** ${\mathrm{MR}}_{3}:T\left(\delta_{s}\left(Im\right)\right)=\delta_{s}(T\left(Im\right))$ ${\mathrm{MR}}_{4}:T\left({ℇ}_{s}\left(Im\right)\right)={\;ℇ}_{s}(T\left(Im\right))$	Where, *T*(.) is the transpose of an image. The image output of transposition followed by dilation and erosion should be similar to image output of dilation and erosion followed by transposition.
**Enhanced Associative Property** ${\mathrm{MR}}_{5}:\;\left(A⊕B\right)⊕C=\left(A⊕C\right)⊕B$	Where, *A* is the input image. *B* and *C* are the structuring elements. Image dilated with structuring element *B* and then dilated with structuring element *C* should give same results when we first dilate the image with structuring element *C* and then dilated with structuring element *B*. This property is specific to dilation as erosion does not fulfill this property.
**Image Translation** ${\mathrm{MR}}_{6}:Trans\left({ℇ}_{s}\left(Im\right)\right)={ℇ}_{s}(Trans\left(Im\right))$	Where, *Trans*(.) is the image translation. The output of image translation followed by erosion should be similar to the output of erosion followed by image translation. This property is not applicable on dilation operation.

**Table 5:** Mutation operators are used in existing techniques and in the proposed framework.

Mutation Operators in Existing Techniques	Mutation Operators in Proposed Framework
ROR—Relational operator replacement LOR—Logical operator replacement	AOD—Unary arithmetic operator deletion AOR—Arithmetic operation replacement LOR—Logical operator replacement ROR—Relational operator Replacement OIL—One iteration loop RIL—Reverse iteration loop SIR—Slice index remove SDL—Statement deletion ZIL—Zero iteration loop

**Table 6:** Sources of source code.

Operation	Source
Sobel Edge Detection	https://towardsdatascience.com/edge-detection-in-python-a3c263a13e03 “(access on 23 April 2021)”
Dilation	https://python.plainenglish.io/image-dilation-explained-easily-e085c47fbac2 “(access on 17 May 2022)”
Erosion	https://medium.com/analytics-vidhya/2d-convolution-using-python-numpy-43442ff5f381 “(access on 17 May 2022)”

**Table 7:** Coverage details.

	Edge Detection Program	Dilation Program	Erosion Program
No. of Test Cases	95	95	95
No. of Statements	41	46	46
No. of Covered Statements	41	46	46
Statement Coverage (%)	100%	100%	100%
No. of Branches	10	12	12
No. of Covered Branches	10	12	12
Branch Coverage (%)	100%	100%	100%

**Table 8:** Effectiveness of Mutation Operators Used in Edge Detection.

Mutation Operators	Number of Mutants Generated	% of Generated Mutants	Mutants Killed by Each MR	FDR of Mutation Operators
AOD	6	3.70%	MR_1_: 6	MR_1_: 100%
			MR_2_: 6	MR_2_: 100%
			MR_3_: 6	MR_3_: 100%
			MR_4_: 6	MR_4_: 100%
AOR	98	60.49%	MR_1_: 73	MR_1_: 74.48%
			MR_2_: 74	MR_2_: 75.51%
			MR_3_: 62	MR_3_: 63.26%
			MR_4_: 62	MR_4_: 63.26%
COI	2	1.23%	MR_1_: 2	MR_1_: 100%
			MR_2_: 2	MR_2_: 100%
			MR_3_: 2	MR_3_: 100%
			MR_4_: 2	MR_4_: 100%
ROR	30	18.51%	MR_1_: 22	MR_1_: 73.33%
			MR_2_: 22	MR_2_: 73.33%
			MR_3_: 22	MR_3_: 73.33%
			MR_4_: 22	MR_4_: 73.33%
OIL	2	1.23%	MR_1_: 1	MR_1_: 50%
			MR_2_: 1	MR_2_: 50%
			MR_3_: 1	MR_3_: 50%
			MR_4_: 1	MR_4_:50%
RIL	2	1.23%	MR_1_: 1	MR_1_: 50%
			MR_2_: 2	MR_2_: 100%
			MR_3_: 0	MR_3_: 0%
			MR_4_: 0	MR_4_: 0%
SIR	4	2.46%	MR_1_: 4	MR_1_: 100%
			MR_2_: 4	MR_2_: 100%
			MR_3_: 4	MR_3_: 100%
			MR_4_: 4	MR_4_: 100%
SDL	16	9.87%	MR_1_: 13	MR_1_: 81.25%
			MR_2_: 13	MR_2_: 81.25%
			MR_3_: 13	MR_3_: 81.25%
			MR_4_: 13	MR_4_: 81.25%
ZIL	2	1.23%	MR_1_: 2	MR_1_: 100%
			MR_2_: 2	MR_2_: 100%
			MR_3_: 2	MR_3_: 100%
			MR_4_: 2	MR_4_: 100%

**Table 9:** Effectiveness of mutation operators used in dilation and erosion operations for proposed MRs.

Mutation Operators	Number of Mutants Generated	% of Generated Mutants	Mutants Killed by Each MR	FDR of Mutation Operators
AOR	95	73.07%	MR_1_: 51	MR_1_: 53.68%
			MR_2_: 50	MR_2_: 52.63%
			MR_3_: 51	MR_3_: 53.68%
			MR_4_: 53	MR_4_: 55.78%
			MR_5_: 56	MR_5_: 58.94%
			MR_6_: 56	MR_6_: 58.94%
COI	4	3.07%	MR_1_: 1	MR_1_: 25%
			MR_2_: 1	MR_2_: 25%
			MR_3_: 1	MR_3_: 25%
			MR_4_: 2	MR_4_: 50%
			MR_5_: 1	MR_5_: 25%
			MR_6_: 2	MR_6_: 50%
ROR	20	15.38%	MR_1_: 3	MR_1_: 15%
			MR_2_: 3	MR_2_: 15%
			MR_3_: 3	MR_3_: 15%
			MR_4_: 6	MR_4_: 30%
			MR_5_: 3	MR_5_: 15%
			MR_6_: 5	MR_6_: 25%
OIL	1	0.76%	MR_1_: 1	MR_1_: 100%
			MR_2_: 0	MR_2_: 0%
			MR_3_: 1	MR_3_: 100%
			MR_4_: 1	MR_4_: 100%
			MR_5_: 0	MR_5_: 0%
			MR_6_: 0	MR_6_: 0%
RIL	1	0.76%	MR_1_: 0	MR1_3_: 0%
			MR_2_: 0	MR1_4_: 0%
			MR_3_: 0	MR1_5_: 0%
			MR_4_: 0	MR1_6_: 0%
			MR_5_: 0	MR1_7_: 0%
			MR_6_: 1	MR1_8_: 100%
SIR	2	1.53%	MR_1_: 1	MR_1_: 50%
			MR_2_: 1	MR_2_: 50%
			MR_3_: 1	MR_3_: 50%
			MR_4_: 1	MR_4_: 50%
			MR_5_: 1	MR_5_: 50%
			MR_6_: 2	MR_6_: 100%
SDL	6	4.61%	MR_1_: 1	MR_1_: 16.6%
			MR_2_: 1	MR_2_: 16.6%
			MR_3_: 1	MR_3_: 16.6%
			MR_4_: 1	MR_4_: 16.6%
			MR_5_: 1	MR_5_: 16.6%
			MR_6_: 1	MR_6_: 16.6%
ZIL	1	0.76%	MR_1_: 1	MR_1_: 100%
			MR_2_: 1	MR_2_: 100%
			MR_3_: 1	MR_3_: 100%
			MR_4_: 1	MR_4_: 100%
			MR_5_: 1	MR_5_: 100%
			MR_6_: 1	MR_6_: 100%

**Table 10:** Fault detection rate of edge detection MRs.

MR	Total No. of Mutants	No. of Killed Mutants	FDR (%)
MR_1_	162	124	76.54%
MR_2_	162	126	77.77%
MR_3_	162	112	69.13%
MR_4_	162	112	69.13%

**Table 11:** Fault detection rate of dilation and erosion MRs.

MR	Total No. of Mutants	No. of Killed Mutants	FDR (%)
R_1_	130	70	53.84%
R_2_	130	68	52.30%
R_3_	130	60	46.15%
R_4_	130	67	51.53%
R_5_	130	52	40.00%
R_6_	130	45	34.61%
R_7_	130	68	52.30%
R_8_	130	65	50.00%

**Table 12:** Fault detection rate of proposed MRs.

MR	Total No. of Mutants	No. of Killed Mutants	FDR (%)
MR_1_	130	58	44.61%
MR_2_	130	56	43.07%
MR_3_	130	58	44.61%
MR_4_	130	64	49.23%
MR_5_	130	62	47.69%
MR_6_	130	67	51.53%

**Table 13:** Statistics of existing technique and proposed framework.

Ref Papers	SUT	No. of Test Cases	Mutation Operator	No. of Mutants Generated	Language
Sim et al. [12]	Edge detection	30	LOR, ROR	31	C
Jameel et al. [14]	Dilation and Erosion	Random selection	ROR	33	Mex C
Proposed framework	Dilation, Erosion, and Edge detection	95	AOD, AOR, OIL, SIR, ZIL, ROR, SDL, COI	162: edge detection 130: dilation and erosion	Python

**Table 14:** Comparison of existing technique and proposed framework.

MR	FDR by Sim et al. Technique [12]	FDR by Proposed Framework
MR_1_	45%	76.54%
MR_2_	77%	77.77%
MR_3_	68%	69.13%
MR_4_	45%	69.13%

**Table 15:** Comparison of existing technique and proposed framework.

MR	FDR by Jameel et al. Technique [14]	FDR by Proposed Framework
R_1_	15%	53.84%
R_2_	58%	54.30%
R_3_	97%	46.15%
R_4_	51%	51.53%
R_5_	58%	40.00%
R_6_	18%	34.61%
R_7_	73%	52.30%
R_8_	21%	50.00%

**Table 16:** Mutants Generated Against Each Mutation Operator.

Mutation Operators	No. of Mutants Generated
AOR	95
COI	4
ROR	20
OIL	1
RIL	1
SIR	2
SDL	6
ZIL	1

## Data Availability

The dataset used in this research is public and is taken from https://www.kaggle.com/datasets/abhranta/brain-tumor-detection-mri?resource=download (accessed on 22 February 2024).
